# Advanced Predictive Modeling of Concrete Compressive Strength and Slump Characteristics: A Comparative Evaluation of BPNN, SVM, and RF Models Optimized via PSO

**DOI:** 10.3390/ma17194791

**Published:** 2024-09-29

**Authors:** Xuefei Chen, Xiucheng Zhang, Wei-Zhi Chen

**Affiliations:** 1 School of Civil Engineering, Putian University, Putian 351100, China; 2 Engineering Research Center of Disaster Prevention and Mitigation of Southeast Coastal Engineering Structures (JDGC03), Fujian Province University, Putian 351100, China; 3 Faculty of Innovation Engineering, Macau University of Science and Technology, Avenida Wai Long, Taipa, Macao 999078, China

**Keywords:** concrete, compressive strength, slump value

## Abstract

This study presents the development of predictive models for concrete performance, specifically targeting the compressive strength and slump value, utilizing the quantities of individual raw materials in the concrete mix design as input variables. Three distinct machine learning approaches—Backpropagation Neural Network (BPNN), Support Vector Machine (SVM), and Random Forest (RF)—were employed to establish the prediction models independently. In the model construction process, the Particle Swarm Optimization (PSO) algorithm was integrated with cross-validation to fine-tune the hyperparameters of each model, ensuring optimal performance. Following the completion of training and modeling, a comprehensive comparison of the predictive accuracy among the three models was conducted, with the aim of selecting the most suitable model for incorporation into an optimized objective function. The findings reveal that among the chosen machine learning techniques, BPNN exhibited superior predictive capabilities for the compressive strength of concrete. Specifically, in the validation set, BPNN achieved a high correlation coefficient (R) of 0.9531 between the predicted and actual outputs, accompanied by a low Root Mean Square Error (RMSE) of 4.2568 and a Mean Absolute Error (MAE) of 2.6627, indicating a precise and reliable prediction. Conversely, for the prediction of the concrete slump value, RF outperformed the other two models, demonstrating a correlation coefficient (R) of 0.8986, an RMSE of 9.4906, and an MAE of 5.5034 in the validation set. This underscores the effectiveness of RF in capturing the complexity and variability inherent in slump behavior. Overall, this research highlights the potential of integrating advanced machine learning algorithms with optimization techniques for enhancing the accuracy and efficiency of concrete performance predictions. The identified optimal models, BPNN for compressive strength and RF for slump, can serve as valuable tools for engineers and researchers in the field of construction materials, facilitating the design of concrete mixes tailored to specific performance requirements.

## 1. Introduction

Concrete, as one of the most widely used construction materials globally, plays a pivotal role in the development of infrastructure projects ranging from bridges and skyscrapers to roads and dams. Its versatility, durability, and cost-effectiveness have made it an indispensable component of modern engineering practices [1,2,3]. However, achieving optimal concrete performance necessitates a precise understanding and control of its various properties, among which compressive strength and slump are two of the most critical [4]. Compressive strength, a measure of the material’s resistance to compressive forces, directly impacts the structural integrity and safety of concrete structures. On the other hand, slump, which refers to the degree of fluidity or consistency of fresh concrete, is essential for ensuring the proper placement, consolidation, and finishing of the material.

Traditionally, the prediction of concrete properties relied heavily on empirical formulas and laboratory testing, which are often time-consuming, expensive, and subject to variations in test conditions and operator skill [2,5]. Furthermore, the complex interactions among the various raw materials and additives in the concrete mix, as well as environmental factors such as temperature and humidity, further complicate the prediction process. Consequently, there is a growing need for more efficient and accurate methods for predicting concrete properties.

In recent years, the advent of machine learning (ML) has revolutionized various fields, including materials science and engineering. ML algorithms, capable of learning from data and making predictions or decisions without being explicitly programmed, have shown immense potential in addressing complex problems that are difficult to model using traditional analytical methods [6,7,8]. By leveraging the vast amounts of data generated in the construction industry, ML models can be trained to identify patterns and relationships between the input variables (e.g., raw material quantities, mixing conditions) and the output variables (e.g., compressive strength, slump) of concrete [9].

This paper aims to contribute to the growing body of research on the application of ML in concrete performance prediction by investigating three popular ML algorithms: Backpropagation Neural Network (BPNN) [10], Support Vector Machine (SVM) [11], and Random Forest (RF) [8]. Each of these algorithms has unique strengths and has been successfully applied in various domains, but their performance in predicting concrete properties remains relatively unexplored. By comparing the predictive accuracy of these algorithms, this study seeks to identify the most suitable model for optimizing the design of concrete mixes tailored to specific performance requirements.

The background and motivation of this study is addressed below. The importance of accurately predicting concrete properties cannot be overstated. Compressive strength, for instance, is a crucial parameter that determines the load-bearing capacity of concrete structures [12]. Underestimating the strength can lead to premature failure and safety hazards, while overestimating it can result in unnecessary material waste and increased costs. Similarly, achieving the desired slump is essential for ensuring the workability and quality of fresh concrete. A slump that is too high or too low can adversely affect the placement, consolidation, and finishing of the material, ultimately compromising the overall performance of the structure [13].

Despite the significance of these properties, traditional prediction methods often suffer from limitations such as lack of precision, reproducibility, and scalability. For example, empirical formulas are based on limited experimental data and may not accurately capture the complex interactions among the various factors affecting concrete properties [14]. Laboratory testing, while more accurate, is resource-intensive and time-consuming, making it impractical for routine use in the design and construction process.

In contrast, ML algorithms offer several advantages over traditional methods. Firstly, they can handle large and complex datasets, identifying patterns and relationships that may be difficult or impossible to discern using analytical methods. Secondly, ML models can be trained on historical data, allowing them to learn from past experiences and make predictions based on new, unseen data. Thirdly, ML algorithms can be continuously improved and refined as more data are collected, enhancing their predictive accuracy over time [2].

The primary objective of this study is to develop and evaluate predictive models for concrete compressive strength and slump using the BPNN, SVM, and RF algorithms. Specifically, the study aims to (1) develop predictive models utilizing the quantities of raw materials in the concrete mix design as input variables to train BPNN, SVM, and RF models for predicting compressive strength and slump. (2) Optimize model parameters by employing the Particle Swarm Optimization (PSO) algorithm in conjunction with cross-validation to tune the hyperparameters of each model, ensuring optimal performance. (3) Compare model performance by evaluating the predictive accuracy of the three models using relevant performance metrics (e.g., correlation coefficient, RMSE, MAE) and selecting the most suitable model for each property. (4) Discuss implications and limitations after analyzing the results and discuss the implications of the findings for the design and construction of concrete structures. Additionally, potential limitations and directions for future research will be identified.

The contributions of this study are indeed multifaceted and significant. Firstly, it offers invaluable insights into the realm of applying machine learning (ML) algorithms for predicting concrete properties. This endeavor fills a notable gap in the existing literature, where the potential of ML in this specific domain has been largely unexplored. By demonstrating the effectiveness of various ML models in accurately predicting key properties of concrete, such as compressive strength and durability, this study paves the way for future research and practical applications in the construction industry.

Secondly, the study identifies the most suitable model among the tested ML algorithms for predicting concrete properties. This finding is of paramount importance, as it not only simplifies the decision-making process for practitioners but also ensures that the chosen model offers the optimal balance between accuracy and computational efficiency. The identification of the optimal model, along with its comprehensive evaluation and comparison with other candidates, provides a solid foundation for the development of more advanced and tailored ML solutions for the construction sector.

In summary, this study not only advances the scientific understanding of ML’s applicability in predicting concrete properties but also presents a practical solution that can be readily adopted by industry professionals. Its dual contributions—filling a knowledge gap and providing a practical guideline for model selection—underscore the significance and impact of this research.

## 2. Methodology for Establishing Concrete Performance Prediction Models

In this study, the machine learning-based performance prediction model is established by utilizing the cubic usage of individual raw materials in concrete mix proportions as inputs and the corresponding standard compressive strength and slump values as outputs. Initially, three machine learning models are fine-tuned and trained through the selection of appropriate hyperparameters. Subsequently, the prediction accuracy of these models is compared to determine the optimal concrete performance prediction model, which will be integrated into the mix proportion optimization framework.

### 2.1. Input and Output Variables of the Prediction Models

(1) Input Variables

Drawing upon previous research, factors significantly influencing the output variables are typically selected as inputs for machine learning models. When constructing a concrete performance prediction model, two primary approaches for selecting input variables exist. The first approach involves directly utilizing the quantities of each raw material in the concrete mix as input variables, resulting in a model that is intuitive and physically meaningful [15]. The second approach employs parameters such as the water–binder ratio and sand ratio as inputs, which reduces the number of variables and enhances modeling efficiency [16].

Given the necessity to establish a relationship between input variables and unit cost, directly incorporating the quantities of individual raw materials as inputs facilitates the formulation of the optimization model’s objective function. Furthermore, relevant research indicates that the first approach yields higher prediction accuracy for concrete slump. Hence, this study adopts the quantities of seven raw materials per cubic meter of concrete—cement content (CC), fly ash content (CFLA), mineral powder content (CMP), fine aggregate content (CFA), coarse aggregate content (CCA), water content (CW), and superplasticizer content (CSP)—as input variables for establishing the concrete performance prediction models.

(2) Output Variables

The output variables chosen for the machine learning models in this study are the cube compressive strength (UCS) and slump, which serve as the predictive outputs.

### 2.2. Sample Data

#### 2.2.1. Overview of Training Sample Data

**Figure 1:**
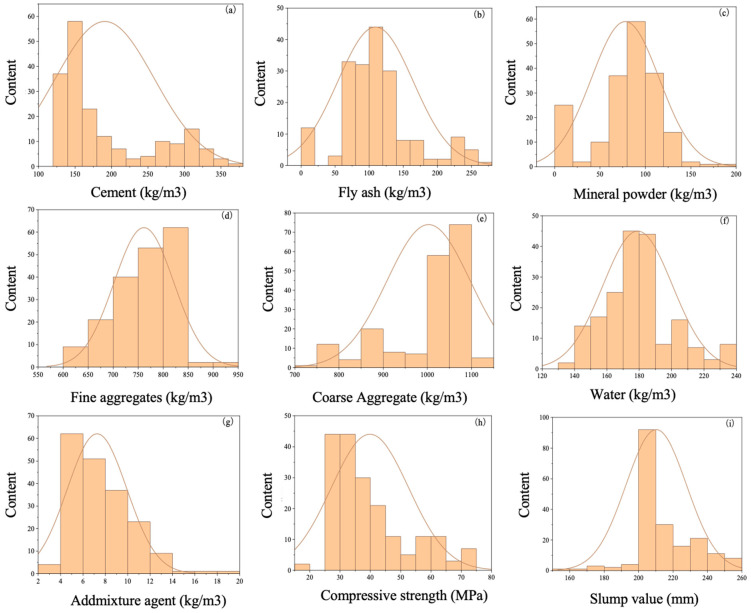
Statistical histogram of sample data: (**a**) statistics chart of C_C_; (**b**) statistics chart of C_FLA_; (**c**) statistics chart of C_MP_; (**d**) statistics chart of C_FA_; (**e**) statistics chart of C_CA_; (**f**) statistics chart of C_W_; (**g**) statistics chart of C_SP_; (**h**) statistics chart of UCS; (**i**) statistics chart of slump.

The primary data source for this study is a commercial concrete company. After filtering out duplicate and conflicting data, a total of 189 concrete mix proportion records, along with their corresponding compressive strength and slump data, are utilized as the overall training sample for the machine learning models. Table 1 presents statistical summaries of the input and output variables within the comprehensive training sample set. Figure 1 illustrates the statistical histograms of each input and output variable within the training sample set, showcasing the distribution of raw material quantities in the mix proportions and the corresponding distributions of compressive strength and slump values.

#### 2.2.2. Preprocessing of Training Data

Prior to utilizing sample data for training machine learning models, it is imperative to subject the data to normalization [17]. This process involves scaling the input and output values to fall within the range of [−1, 1], as per Equation (1) [18]. The objective behind this normalization is twofold: firstly, to satisfy the requirements of the activation function employed in the model, and secondly, to enhance the convergence speed of the training process. Normalization ensures that data features are on a comparable scale, which is crucial for algorithms that rely on distance calculations or weight updates during optimization, thereby fostering improved performance and stability [19].

By scaling the data to a uniform range, the model becomes less sensitive to the magnitude of the input values and can focus more on their relative importance. This is particularly advantageous in scenarios where different features exhibit vastly different scales, as it prevents any single feature from dominating the learning process. Additionally, normalization can also help to mitigate issues related to vanishing or exploding gradients, a common challenge in deep learning models, by ensuring that gradients remain within a manageable range during backpropagation. In summary, preprocessing the training data through normalization is a crucial step towards enhancing the performance, convergence speed, and overall stability of machine learning models, particularly in the context of utilizing them for complex prediction or classification tasks [20].

(1) $$y=\frac{\left(y_{max}-y_{min}\right)\left(x_{max}-x_{min}\right)}{x_{max}-x_{min}}+y_{min}$$

where x and y represent the input and output values, respectively, for the normalization calculation. The target range for normalization is denoted as [*y_min_*, *y_max_*], while [*x_min_*, *x_max_*] represents the range of the input values that are being normalized.

### 2.3. Model Evaluation Approaches

A well-performing machine learning prediction model necessitates not only a high degree of goodness-of-fit to the training data but also robust generalization capabilities [21]. To ensure that the established prediction model meets operational requirements, it is imperative to evaluate its predictive performance through appropriate assessment methodologies. The evaluation process involves several critical steps that enhance both the academic rigor and logical coherence of the analysis [22]. Firstly, the choice of evaluation metrics is paramount, as they must accurately reflect the model’s ability to generalize beyond the training data. Commonly used metrics include accuracy, precision, recall, F1-score for classification tasks, Mean Square Error (MSE), Root Mean Square Error (RMSE), and Mean Absolute Error (MAE) for regression problems. These metrics provide quantitative insights into the model’s performance, enabling researchers to compare and select the most suitable model.

Furthermore, the adoption of cross-validation techniques, such as k-fold cross-validation, is essential to mitigate the risk of overfitting. By dividing the dataset into k distinct subsets, training the model on k−1 subsets, and validating it on the remaining subset, cross-validation ensures that the model’s performance is evaluated on a wide range of data points, thus providing a more reliable estimate of its generalization capabilities [23]. Moreover, the use of independent test sets is crucial for assessing the model’s performance in real-world scenarios. A test set, separate from both the training and validation sets, allows for an unbiased evaluation of the model’s predictive accuracy on unseen data. This step ensures that the model’s performance is not artificially inflated due to the model’s overfitting to the training or validation data.

Lastly, the evaluation process should also involve a thorough analysis of the model’s limitations and potential biases. This includes examining the model’s performance across different subsets of the data, such as those based on specific features or categories, to identify any disparities or inconsistencies. By identifying and addressing these limitations, researchers can further refine and improve the model, enhancing its overall robustness and reliability. A comprehensive model evaluation approach that incorporates appropriate metrics, cross-validation techniques, independent test sets, and a rigorous analysis of limitations and biases is essential for ensuring that the established prediction model meets the desired standards of academic rigor, logical coherence, and practical applicability [24].

The prevalent evaluation methodologies in machine learning encompass the bootstrap method, holdout method, and cross-validation method. Their fundamental premise revolves around segregating a portion of the dataset to gauge the model’s generalization capability.

(1) Bootstrap Method

The bootstrap method relies on resampling with replacement. For an original dataset D comprising n samples, n random samples are drawn with replacement, forming the training set D’. Due to the replacement, some samples remain unselected, and these unsampled instances constitute the validation set, used to evaluate the model’s predictive performance.

The probability of a sample not being selected in n trials is given by:

(2) $$P\left(Never\;selected\;once\right)={\left(1-\frac{1}{n}\right)}^{n}$$

(3) $$\underset{n\to \infty }{\lim}{\left(1-\frac{1}{n}\right)}^{n}=\frac{1}{e}\approx 0.368$$

This implies that roughly 36.8% of the total samples are utilized as the test set in the bootstrap method, while the training set retains the same size as the original dataset. Although the bootstrap method is advantageous when the training sample size is limited, the altered distribution of D’ compared to the original D introduces an inherent estimation bias in the evaluation results.

(2) Holdout Method

The holdout method involves partitioning the original dataset D directly into two mutually exclusive sets: a training set S for model training and parameter tuning, and a test set T for validating the model’s predictions.

(4) D=S∪T and S∩T=∅

Typically, two-thirds or four-fifths of the total samples n are allocated to the test set T. A notable drawback of the holdout method is its sensitivity to the specific partition, leading to substantial randomness in the evaluation outcomes. To mitigate this, multiple holdout evaluations are often conducted, and the average error is computed to reduce the impact of chance. Nevertheless, the holdout method also entails utilizing only a subset of the dataset for training, thereby introducing non-negligible estimation bias.

(3) Cross-Validation Method

Stemming from the holdout method, cross-validation divides the original training set into v roughly equal-sized, mutually exclusive subsets, known as v-fold cross-validation:

(5) $$D=D_{1}\cup D_{2}\cup D_{3}\ldots \cup D_{v}\;and\;D_{i}\cap D_{j}=\emptyset \left(i\neq j\;and\;i,j\leq v\right)$$

**Figure 2:**
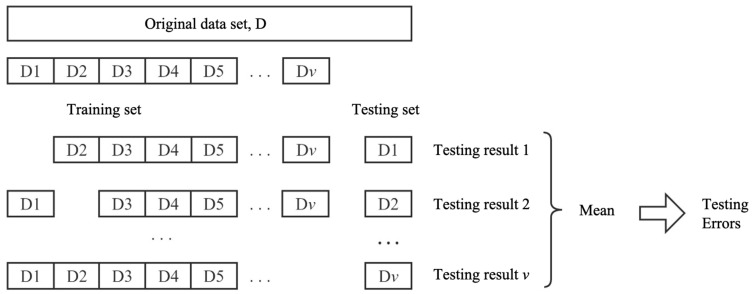
Diagram of cross-validation.

As illustrated in Figure 2, each subset is iteratively designated as the validation set, while the rest serve as the training set. The average of the v evaluation outcomes is then taken as the final evaluation metric. Among the three methods, cross-validation, with an appropriately chosen number of folds, minimizes the estimation bias between the model evaluation results and the true model performance. Consequently, this study employs cross-validation as the primary approach for assessing the predictive performance of the model.

### 2.4. Evaluation Metrics for Model Performance

In this study, the Pearson correlation coefficient (R), Root Mean Square Error (RMSE), and Mean Absolute Error (MAE) are employed as the primary assessment criteria for evaluating the predictive performance of the machine learning models. These metrics provide a comprehensive evaluation framework that encapsulates both the accuracy and the correlation strength between predicted and observed values.

The Pearson correlation coefficient (R) (see Equation (6)) specifically quantifies the degree of linear relationship between the predicted and actual values [25]. Its value ranges from −1 to 1, where a positive R indicates a positive correlation and a negative R signifies a negative correlation. Furthermore, the closer the absolute value of R is to 1, the stronger the correlation between the variables [26].

(6) $$R=\frac{\sum_{i=1}^{n}\left(y_{i}^{*}-\bar{y^{*}}\right)\left(y_{i}-\bar{y}\right)}{\sqrt{\sum_{i=1}^{n}{\left(y_{i}^{*}-\bar{y^{*}}\right)}^{2}}\sqrt{\sum_{i=1}^{n}{\left(y_{i}-\bar{y}\right)}^{2}}}$$

Moreover, RMSE and MAE are employed to assess the model’s predictive accuracy by quantifying the average deviation of predicted values from their actual counterparts [27]. RMSE, being a squared error metric, is sensitive to outliers and provides a more stringent measure of the prediction errors. In contrast, MAE, as an absolute error metric, is less affected by extreme deviations and offers a more robust assessment, particularly for skewed error distributions. Both RMSE and MAE serve as valuable indicators of the model’s overall predictive precision, complementing the insights gained from the correlation analysis. Collectively, these evaluation metrics enable a multifaceted assessment of the machine learning models, ensuring that the chosen model not only exhibits a strong correlation with the ground truth but also achieves high predictive accuracy, thereby enhancing the reliability and interpretability of the model’s predictions [28].

(7) $$RMSE=\sqrt{\frac{1}{n}\sum_{i=1}^{n}{\left(y_{i}^{*}-y_{i}\right)}^{2}}$$

(8) $$MAE=\frac{1}{n}\sum_{i=1}^{n}\left|y_{i}^{*}-y_{i}\right|$$

### 2.5. Determination of Model Hyperparameters

In the realm of machine learning, hyperparameters represent the external configuration variables of a model, which are typically adjusted based on a specific predictive modeling problem. These values cannot be directly estimated from the data but require strategic tuning. While achieving the optimal hyperparameter values for a given problem is often elusive, several approaches, such as rule-of-thumb heuristics, trial-and-error methods, and optimization algorithms, have been employed to identify the most suitable hyperparameters for a machine learning model [29].

Hyperparameter optimization constitutes a “black-box optimization” problem, where only the inputs and outputs are observable during the tuning process [30]. Given the resource-intensive nature of model training (in terms of time, hardware, etc.), it is imperative to adopt “accurate and efficient” optimization strategies. In this study, we leverage both empirical knowledge and experimentation to fine-tune a subset of hyperparameters with narrower ranges while utilizing optimization algorithms for those with broader ranges or whose optimal values are difficult to ascertain through intuition or trial.

#### 2.5.1. Empirical and Experimental Determination of Select Hyperparameters

(1) Hyperparameters for the BPNN Model

The fundamental parameters of the Backpropagation Neural Network (BPNN) model are determined through a combination of authoritative literature references [6,31,32,33] and empirical adjustments based on specific sample training sessions. Table 2 outlines the baseline BPNN parameters, including the use of the Levenberg–Marquardt algorithm (TRAINLM), a widely adopted nonlinear least-squares optimization technique. The hidden layer employs the logistic sigmoid transfer function (logsig, see Equation (9)), while the output layer utilizes the purelin linear function, specified in Equation (10).

(9) $$f\left(x\right)=\frac{1}{1+e^{-x}}$$

(10) y=x

(2) Hyperparameters for the SVM Model

The Support Vector Machine (SVM) model is constructed using the libsvm toolbox, with its parameters determined based on relevant literature and their suitability for the sample modeling in this study [34]. The Radial Basis Function (RBF) is selected as the kernel function, with its mathematical expression given in Equation (11). Additionally, the penalty parameter (p) of the loss function is set to 0.001.

(11) $$K\left(x_{i},y_{j}\right)=e^{-\gamma {\left\|x_{i}-y_{j}\right\|}^{2}}$$

where *x_i_*, *y_j_* represent samples or vectors, γ is the hyperparameter gamma, and $\left\|x_{i}-y_{j}\right\|$ denotes the norm of the vector difference.

(3) Hyperparameters for the RF Model

The Random Forest (RF) model’s primary hyperparameter, the number of variables randomly sampled at each split (mtry), is set according to established literature. The value of mtry is calculated using Equation (12), which ensures an appropriate balance between the number of input features (m) and the complexity of the individual trees. This approach ensures that the RF model is both computationally efficient and effective in handling high-dimensional data. In summary, by strategically combining empirical insights, experimentation, and optimization algorithms, we aim to identify the optimal hyperparameter configurations for our machine learning models, thereby enhancing their predictive performance and reliability.

(12) $$mtry=max\left(floor\left(\frac{m}{3}\right),1\right)$$

#### 2.5.2. Determination of Partial Hyperparameters via Intelligent Optimization Algorithms

**Figure 3:**
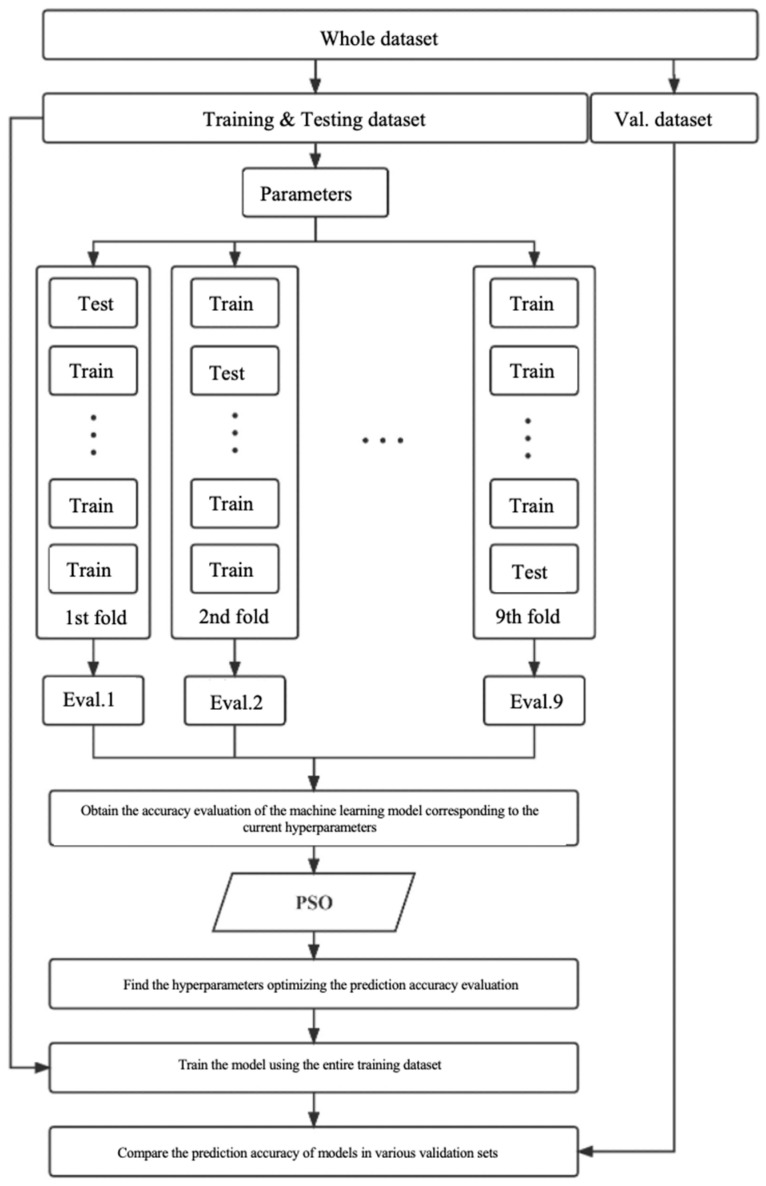
Flow chart of the hyperparameter optimization process.

In this study, grounded on pertinent literature and the size of the sample dataset, we employ the Particle Swarm Optimization (PSO) algorithm in conjunction with a nine-fold cross-validation strategy. This approach aims to optimize selected hyperparameters of prediction models such as Backpropagation Neural Networks (BPNNs), Support Vector Machines (SVMs), and Random Forests (RFs), with the Root Mean Square Error (RMSE) between predicted and actual outputs serving as the primary criterion for assessing model prediction accuracy [35]. The hyperparameter optimization process, as illustrated in Figure 3, proceeds through the following steps:

Step 1: Data Partitioning

Initially, the entire dataset is divided into two distinct parts: a training validation set and a holdout set, according to a predefined ratio. The training validation set is then further uniformly split into nine subsets, each serving a specific role in the subsequent cross-validation process.

Step 2: Cross-Validation and Evaluation

The hyperparameters of the machine learning models are randomly initialized, and an iterative cross-validation process commences. In each iteration, one of the nine subsets is designated as the test set, while the remaining eight are utilized as the training set. This procedure is repeated nine times, ensuring that each subset serves as the test set once. For each iteration, the RMSE between the actual outputs of the test set samples and the corresponding model predictions is calculated. The average of these RMSE values is subsequently computed, providing a comprehensive assessment of the model’s prediction accuracy under the current hyperparameter configuration.

Step 3: Optimization via PSO

Leveraging the PSO algorithm, an iterative search process is initiated to refine the hyperparameter values. At each iteration, the PSO algorithm updates the hyperparameters based on the principles of swarm intelligence, aiming to minimize the average RMSE. The cross-validation process described in Step 2 is repeated with the updated hyperparameters.

Step 4: Convergence and Optimal Hyperparameters

The PSO algorithm continues iterating until it reaches a predefined termination criterion, such as a maximum number of iterations or a satisfactory level of convergence. Upon completion, the optimal hyperparameter values that maximize the prediction accuracy of the machine learning models are identified.

Subsequently, the optimized hyperparameters obtained from PSO are individually incorporated into the BPNN, SVM, and RF models. Each model is retrained using the training validation set and evaluated on the holdout set to assess their prediction accuracies. The model demonstrating the highest prediction accuracy for concrete performance is then selected for subsequent multi-objective optimization of concrete mix proportions. This approach ensures that the selected model is equipped with the most suitable hyperparameters, thereby enhancing its predictive capability and reliability in real-world applications.

In this study, the hyperparameters determined through the application of intelligent optimization algorithms encompass several key aspects: for BPNNs, the number of hidden layers and the individual neuron count within each hidden layer; for SVMs, the gamma value of the kernel function and the penalty parameter (c-value) that controls the trade-off between the margin size and the training error; and for RFs, the number of decision trees constituting the random forest, as well as the minimum number of samples required to split an internal node into a leaf node. Table 3 outlines the predefined search ranges for these hyperparameters corresponding to each machine learning model during the optimization process, drawing upon relevant references [36,37,38] and best practices. This methodical approach ensures that the optimization process comprehensively explores a broad spectrum of hyperparameter configurations, thereby increasing the likelihood of identifying optimal values that significantly enhance the predictive performance and robustness of the models. By fine-tuning these hyperparameters, we aim to harness the full potential of BPNNs, SVMs, and RFs in accurately predicting concrete properties, ultimately facilitating the multi-objective optimization of concrete mix designs.

## 3. Results and Discussion

### 3.1. Establishment of Concrete Mix Proportion—Compressive Strength Prediction Model

This section outlines the development of a predictive model that utilizes the quantities of seven raw materials—cement, fly ash, mineral powder, fine aggregate, coarse aggregate, water, and water-reducing admixture—per cubic meter of concrete as input variables, with the compressive strength of the resulting concrete serving as the output variable. This model aims to establish a robust relationship between the mix proportion and the critical mechanical property of concrete.

#### 3.1.1. Hyperparameter Tuning of Machine Learning Models

To refine the performance of the machine learning (ML) models employed in this study, a comprehensive hyperparameter optimization process was conducted leveraging Particle Swarm Optimization (PSO) in conjunction with nine-fold cross-validation. Specifically, the PSO algorithm was configured with a swarm size of 20 particles, an iteration limit of 40 cycles, cognitive and social coefficients (c1 and c2) both set to 2, and a velocity range constrained within [−1, 1]. The fitness function was designed to evaluate the models based on the Root Mean Square Error (RMSE) obtained from the nine-fold cross-validation procedure, ensuring a rigorous and unbiased assessment.

**Figure 4:**
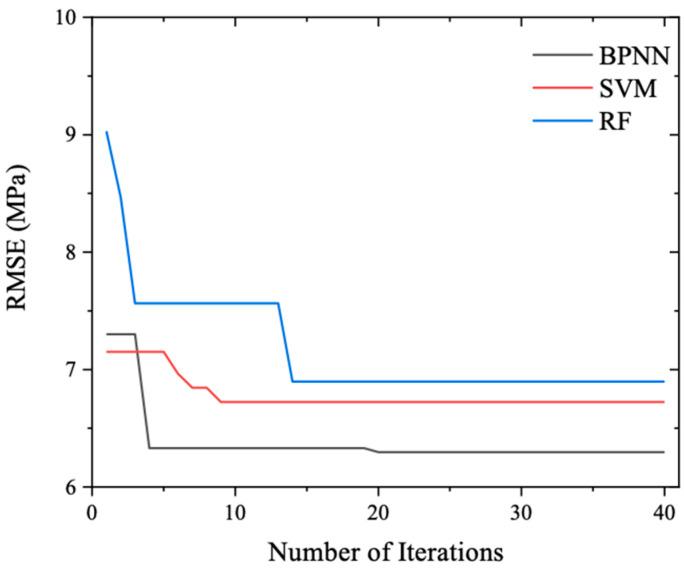
Fitness versus iteration during hyperparameter tuning.

Figure 4 illustrates the evolution of the global best fitness values across iterations for the various ML models. As the iteration count increases, a discernible downward trend is observed in the fitness values of all models, demonstrating the efficacy of the PSO algorithm in swiftly and effectively adjusting the hyperparameters to enhance prediction accuracy. After 40 iterations, the fitness curves for the three models—Backpropagation Neural Network (BPNN), Support Vector Machine (SVM), and Random Forest (RF)—plateau, indicating that the optimization process has converged to near-optimal solutions. Notably, BPNN achieves the lowest fitness value, followed by SVM, with RF exhibiting the highest fitness, suggesting that BPNN may offer the best predictive performance under the optimized hyperparameters. This hyperparameter-tuning process not only underscores the importance of careful configuration in harnessing the full potential of ML models but also provides a rigorous framework for selecting the most suitable model for the specific task of predicting concrete compressive strength based on mix proportion.

Numerical analysis was incorporated, and Table 4 records the global best fitness values obtained from optimizing the hyperparameters of the BPNN, SVM, and RF compressive strength prediction models using the Particle Swarm Optimization (PSO) algorithm. Furthermore, Table 5 documents the optimal hyperparameters corresponding to these global best fitness values. Upon examination of these tables, it becomes evident that upon completion of the hyperparameter optimization process, the Root Mean Square Error (RMSE) values between the predicted and actual compressive strength outputs on the test set follow the order of RMSEBPNN < RMSESVM < RMSERF. This observation signifies that, under identical optimization conditions, the BPNN model exhibits superior prediction accuracy for concrete compressive strength when evaluated on the test set samples.

The superiority of BPNN in this context can be attributed to its ability to capture complex nonlinear relationships within the data, enabled by its flexible network architecture and adaptive learning mechanisms [39]. By adjusting the weights and biases of its neurons during training, BPNN can effectively model the intricate interplay between the various mix proportion components and their influence on the final compressive strength of the concrete. Consequently, when equipped with the optimal hyperparameters identified through the PSO-based optimization, BPNN demonstrates a higher level of predictive performance, underscoring its suitability for precision engineering applications, where accurate predictions of material properties are crucial.

#### 3.1.2. Selection of Concrete Compressive Strength Prediction Models

To further evaluate the performance of the optimized models, the identified optimal hyperparameters were individually incorporated into the BPNN, SVM, and RF frameworks. Subsequently, the training and testing sets, which were originally utilized for hyperparameter optimization, were merged to form a comprehensive new training set. This consolidated dataset was then employed to retrain each model. Upon completion of the retraining process, the predictive accuracy of the models was assessed separately for both the training and validation sets. Metrics such as Root Mean Square Error (RMSE), R-squared (R), and Mean Absolute Error (MAE) were calculated for both sets, allowing for a comparative analysis of the models’ performances. The results of this comparative analysis are systematically recorded in Table 6, providing a comprehensive view of the models’ efficacies in capturing the complex relationships within the data and predicting the concrete’s compressive strength with precision.

**Figure 5:**
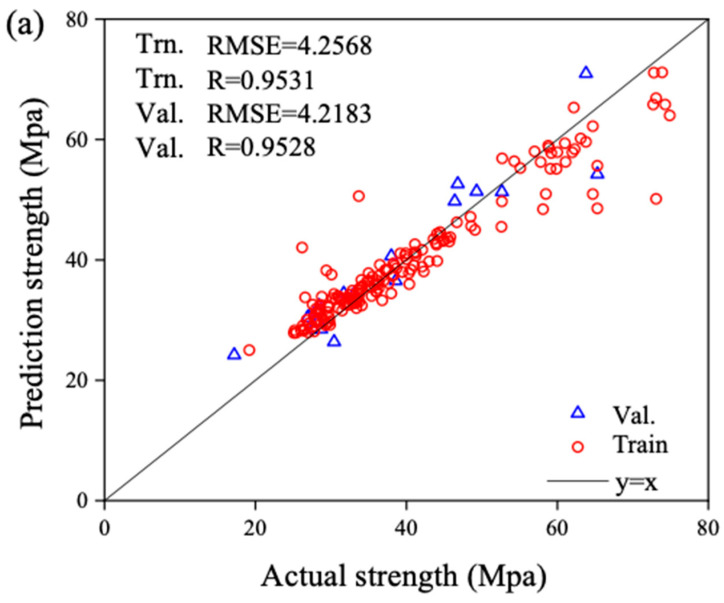

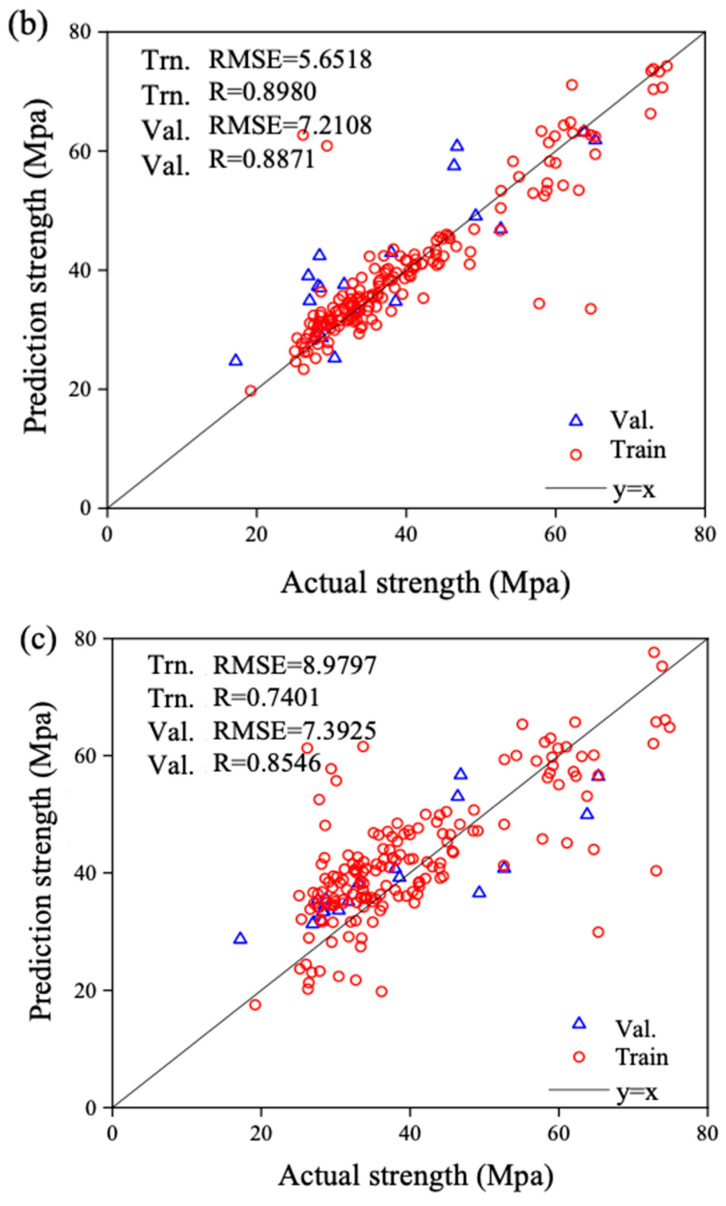
Predicted versus actual UCS values on the training and validation sets using the ML model: (**a**) predicted performance of BPNN; (**b**) predicted performance of SVM; (**c**) predicted performance of RF.

Figure 5 presents a comparative analysis of the actual compressive strength values versus the predicted outputs of the models. Specifically, subgraph (a) contrasts the predicted strength from BPNN against the actual strength, subgraph (b) shows the comparison for SVM, and subgraph (c) depicts the same for RF. The ideal curve y = x serves as a benchmark, and the proximity of data points to this curve illustrates the goodness-of-fit for the training set samples and the predictive accuracy for the validation set samples across the three models.

Upon examining the distribution of data points in all three figures vis-à-vis the ideal curve, it becomes evident that subgraph (a) exhibits a tight clustering of points around the ideal curve, indicating the highest predictive accuracy of BPNN. Subgraph (b) displays a slightly more dispersed pattern yet still relatively close to the ideal curve, suggesting SVM’s secondary predictive accuracy. Conversely, subgraph (c) reveals the most dispersed distribution, signifying the lowest predictive accuracy among the three models for concrete strength performance.

A closer inspection of the training and validation sets within each figure reveals that in subgraphs (a) and (b), the data points representing the training set are closer to the ideal curve than those of the validation set, underscoring the superior fitting performance of BPNN and SVM on the training samples compared to their predictive accuracy on the validation set. However, in subgraph (c), the validation set data points are closer to the ideal curve than the training set, indicating that the RF model exhibits a lower fitting quality on the training samples but a better predictive accuracy on the validation set.

Comparing the goodness-of-fit across the training and validation sets for the three models, it is observable that in regions with denser and more abundant training samples (strength range of 30–50 MPa), the validation set samples tend to align more closely to the ideal curve. Conversely, in areas with sparse and scattered training samples (strength of less than 30 MPa or greater than 50 MPa), the validation set samples deviate further from the ideal curve, emphasizing the influence of sample size distribution on predictive accuracy. Adequate training samples lead to improved predictive performance, whereas scarcity can diminish model accuracy.

**Figure 6:**
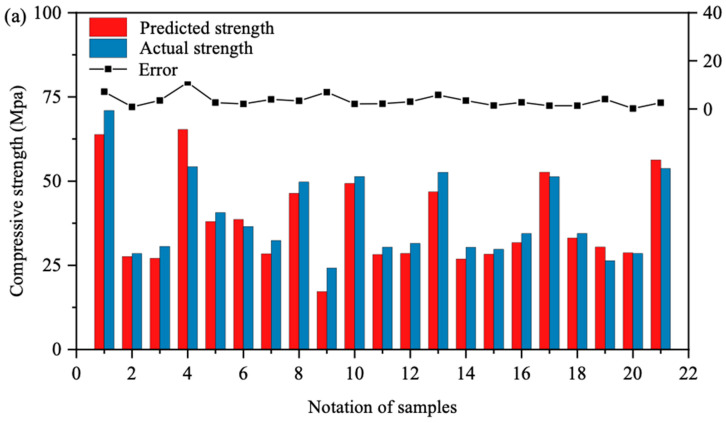

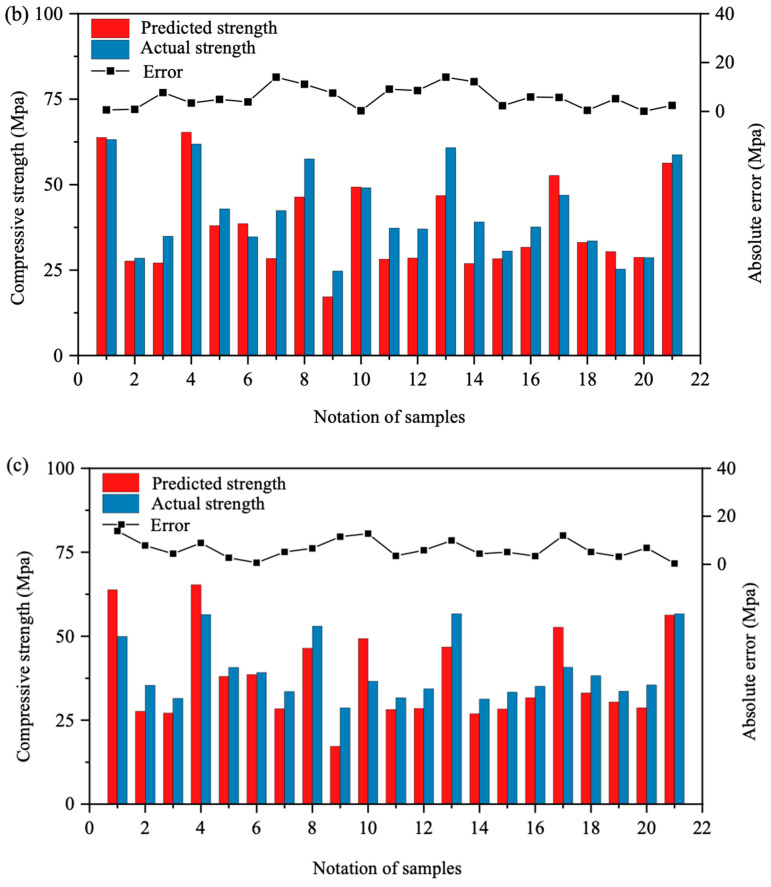
Error analyses between the predicted value and actual value of UCS of the ML model: (**a**) BPNN model prediction error; (**b**) SVM model prediction error; (**c**) RF model prediction error.

Figure 6 provides an error analysis of the model predictions, with Figure 6a focusing on BPNN’s prediction errors for the validation set, Figure 6b on SVM, and Figure 6c on RF. The histograms represent the predicted and actual compressive strengths in the validation set, while the line graphs depict the absolute errors between model outputs and actual values. Analysis of these figures indicates that in Figure 6a, the height difference between the red and blue histograms is minimal, with a relatively low error curve, signifying minimal absolute error between BPNN’s predictions and the actual values. In Figure 6b, the histogram difference is slightly larger, resulting in a higher error curve compared to Figure 6a. Lastly, Figure 6c exhibits the largest histogram difference and the highest error curve, indicating the largest absolute errors for RF. In summary, BPNN demonstrates the smallest absolute errors in the validation set, followed by SVM, with RF exhibiting the largest errors.

**Figure 7:**
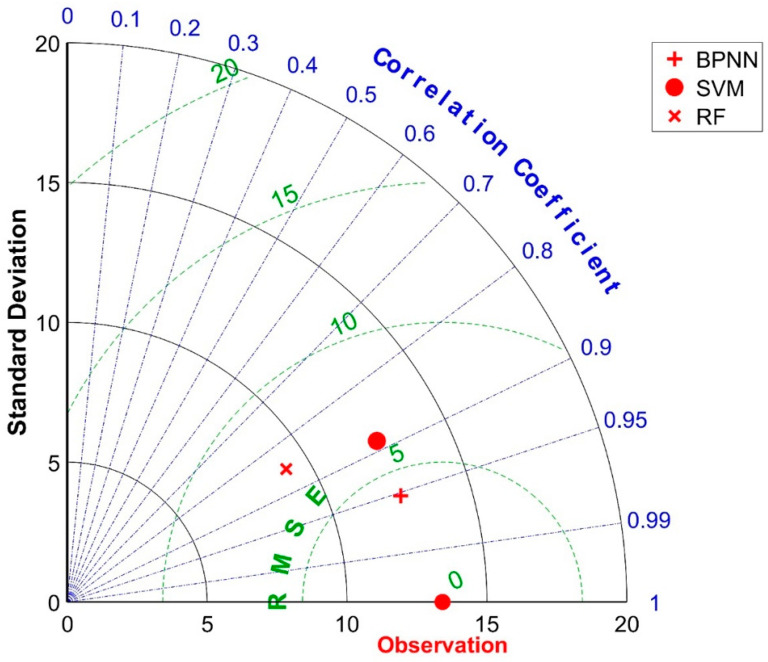
Taylor diagram comparison of the machine learning model validation set’s prediction performance.

To facilitate a more definitive comparison and subsequent selection of the optimal strength prediction model in terms of predictive performance, this study employs Taylor diagrams for model evaluation and selection. In these diagrams, scatter points represent machine learning models, with arcs centered at the origin signifying the standard deviation (SD), radial lines indicating the correlation coefficient (R), and arcs centered on an observation point representing the root Mean Square Error (RMSE). Figure 7 presents the Taylor diagram for evaluating the accuracy of the three predictive models on the validation set, offering a straightforward visual comparison of their performances with optimal hyperparameters. The observation point in the diagram is positioned at the ideal scenario of R = 1 and RMSE = 0, while the standard deviation SD = 13.4138 corresponds to the standard deviation of the compressive strength samples in the validation set.

By analyzing the positional relationships between the scatter points and radial lines in the diagram, it can be inferred that all three models exhibit R values above 0.8 for the validation set, indicative of strong correlations between their predicted outputs and actual outputs. Comparing the angles formed between the rays extending from the origin to each scatter point and the vertical axis reveals the hierarchy of correlation coefficients: R_BPNN > R_SVM > R_RF. Furthermore, examining the straight-line distances between the scatter points and the observation point indicates that BPNN has the shortest distance, followed by SVM, and then RF with the farthest, signifying RMSE_BPNN < RMSE_SVM < RMSE_RF. Consequently, BPNN is chosen for establishing the concrete strength prediction model to be utilized in the multi-objective optimization of mix proportions. The trained BPNN strength prediction model is henceforth denoted as NET.

### 3.2. Establishment of Concrete Mix Proportion—Slump Prediction Model

Given the distinct mapping relationships between slump, compressive strength, and concrete mix proportions, the optimal hyperparameters identified for strength prediction models may not necessarily translate well to slump prediction. Consequently, the establishment of a slump prediction model necessitates reiterating the aforementioned steps, with a focus on identifying the hyperparameter values that yield the most optimal performance specifically for predicting concrete slump. Subsequently, a comparative analysis is conducted to select the model exhibiting the best predictive capabilities.

#### 3.2.1. Fine-Tuning of Machine Learning Model Hyperparameters

Analogous to the approach for strength prediction, the hyperparameters of the slump prediction models are fine-tuned using Particle Swarm Optimization (PSO) in conjunction with nine-fold cross-validation. Here, the PSO algorithm is configured with a swarm size of 20, an iteration count of 40, cognitive and social coefficients (c1 and c2) both set to 2, and a velocity range within [−1, 1]. The fitness function adopted for evaluating the performance is the Root Mean Square Error (RMSE) obtained from the nine-fold cross-validation.

**Figure 8:**
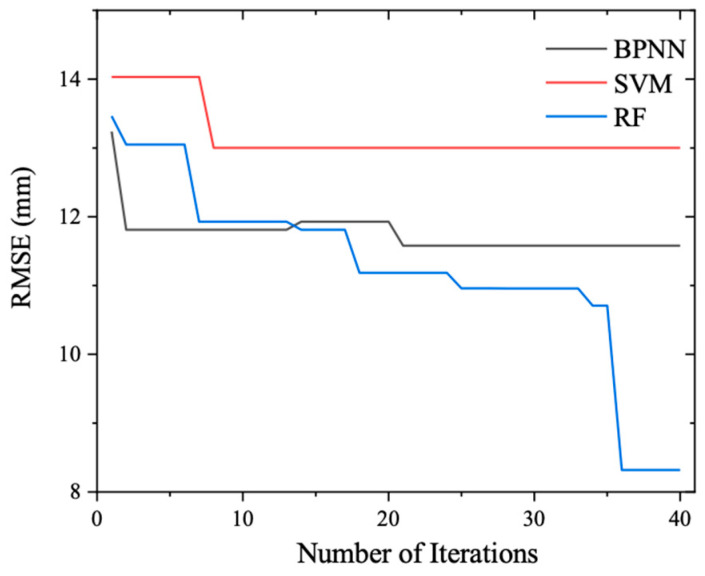
Fitness versus iteration during hyperparameter tuning.

The evolution of the global best fitness values across iterations, as depicted in Figure 8, reveals that initially, the fitness values of the BPNN, SVM, and RF slump prediction models are relatively close. However, as the iterations progress, the fitness values decrease to varying degrees for each model. By the 40th iteration, the fitness values stabilize, with RF exhibiting the lowest fitness, followed by BPNN, and with SVM attaining the highest fitness. Table 7 summarizes the global best fitness values (i.e., RMSE) achieved by the BPNN, SVM, and RF models after PSO-based hyperparameter optimization, while Table 8 details the corresponding optimized hyperparameters. A numerical analysis of these results indicates that, on average, the RMSE values in the test set follow the order RMSE_RF < RMSE_BPNN < RMSE_SVM. This underscores the superiority of the RF model, equipped with optimized hyperparameters, in predicting concrete slump under identical optimization conditions. In summary, the dedicated hyperparameter tuning process tailored specifically for slump prediction ensures that the most appropriate model configuration is identified, thereby enhancing the accuracy and reliability of slump predictions in concrete engineering applications.

Upon a comparative analysis, it becomes evident that substantial disparities persist in the optimal hyperparameters utilized for developing intensity prediction models versus slump prediction models, even when employing the same machine learning algorithm. This discrepancy stems from the fundamental fact that alterations in the mapping relationship between input and output variables necessitate corresponding adjustments in the hyperparameters tailored for the machine learning prediction models, even if the input variables remain unchanged. In an academic and logical context, the sensitivity of hyperparameters to the specific prediction task underscores the importance of fine-tuning these parameters for each individual model. While both compressive strength and slump prediction tasks may involve similar sets of input data, the underlying complexities and non-linearities governing the relationships between these inputs and their respective outputs differ significantly. As a result, the optimal hyperparameter configuration that maximizes predictive performance for one task (e.g., compressive strength prediction) may not necessarily translate well to another task (e.g., slump prediction).

Therefore, it is crucial to engage in a systematic process of hyperparameter optimization tailored specifically to each prediction objective. This process involves iteratively exploring the hyperparameter space, evaluating the performance of the model with different parameter combinations, and selecting the set that yields the most accurate and reliable predictions for the task at hand [40]. By doing so, researchers can ensure that the machine learning models they develop are optimally configured to address the unique challenges and nuances of the respective prediction tasks.

#### 3.2.2. Selection of Concrete Slump Prediction Models

To ascertain the most suitable model for predicting concrete slump, the identified optimal hyperparameters were individually incorporated into the BPNN (Backpropagation Neural Network), SVM (Support Vector Machine), and RF (Random Forest) algorithms. Subsequently, these models were trained using the designated training–testing dataset. Upon completion of the training phase, a rigorous evaluation process was conducted to assess the predictive accuracy of each machine learning model, within both the training set and the validation set.

Specifically, the Root Mean Square Error (RMSE), R-value (often referred to as the correlation coefficient or coefficient of determination, R^2^), and Mean Absolute Error (MAE) were calculated for each model across both datasets. These metrics provide comprehensive insights into the performance of the models, offering quantitative measures of precision, correlation strength, and average deviation from the actual values, respectively. The computed RMSE, R, and MAE values were then systematically recorded in Table 9 for further analysis and comparison.

This systematic approach ensures that the selection of the optimal slump prediction model is grounded in rigorous empirical evidence, taking into account not only the predictive accuracy but also the robustness and generalizability of the models across different datasets. By comparing the performance indicators across BPNN, SVM, and RF, researchers can identify the model that best suits the unique characteristics of the concrete slump prediction task, thereby enhancing the academic rigor and practical applicability of the study.

**Figure 9:**
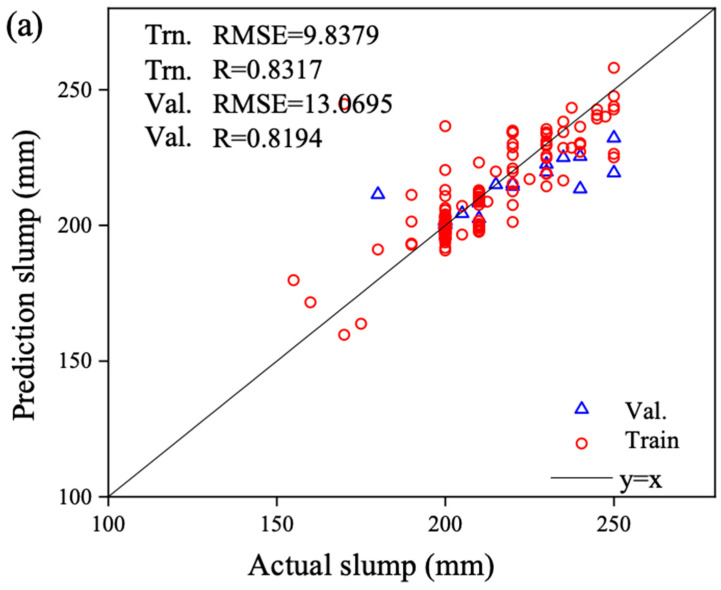

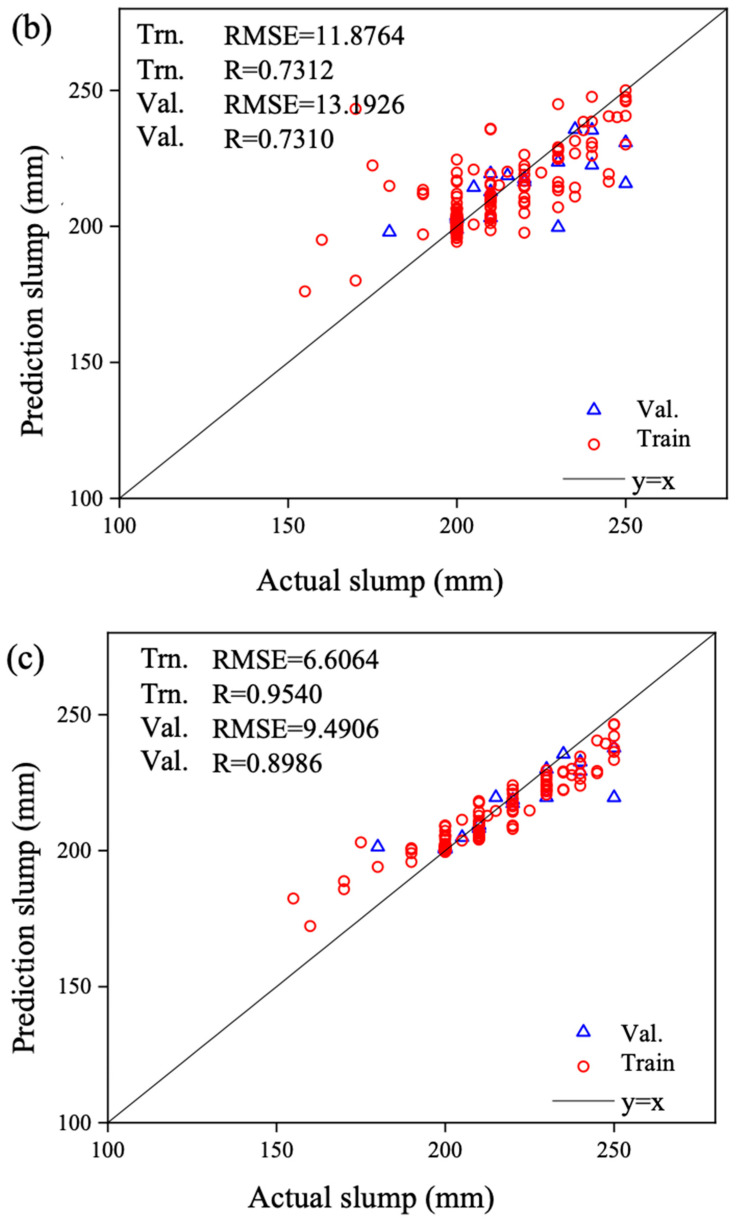
Predicted versus actual slump values on the training and validation sets using the ML model: (**a**) predicted performance of BPNN; (**b**) predicted performance of SVM; (**c**) predicted performance of RF.

Figure 9 presents a comparative analysis of the actual slump values versus the predicted outputs from the models. Subfigure (a) depicts the comparison between the predicted slump from BPNN and the actual slump, (b) shows the comparison for SVM, and (c) illustrates the comparison for RF. The ideal curve, y = x, is overlaid to visually assess the goodness-of-fit for the training set samples and the predictive accuracy for the validation set samples, as indicated by the proximity of data points to this curve.

Upon examining the distribution of data points around the ideal curve in all three subfigures, it is evident that subgraph (c) exhibits a tight clustering of points near the ideal line, while those in subgraphs (b) and (a), to a lesser extent, are more dispersed. This indicates that RF achieves the highest predictive accuracy for concrete slump properties, whereas SVM and BPNN demonstrate lower levels of accuracy.

Comparing the distribution of training and validation set data points within each subfigure, it becomes clear that the data points representing the training set are consistently closer to the ideal curve than those of the validation set in subgraphs (a), (b), and (c). This underscores that the goodness-of-fit for the training set samples is superior to the predictive accuracy for the validation set samples across BPNN, SVM, and RF slump prediction models.

Analyzing the goodness-of-fit for both training and validation sets across the three models, we observe that in regions where the training set is abundant and in high density (i.e., slump values of between 200 and 230 mm), the validation set samples tend to adhere more closely to the ideal curve. Conversely, in areas with sparse and scattered training samples (slump values of less than 200 mm or greater than 230 mm), the validation set samples deviate further from the ideal curve. This suggests that the distribution of sample sizes significantly impacts predictive accuracy, with better performance in intervals where training samples are relatively abundant and decreased accuracy in underrepresented regions.

**Figure 10:**
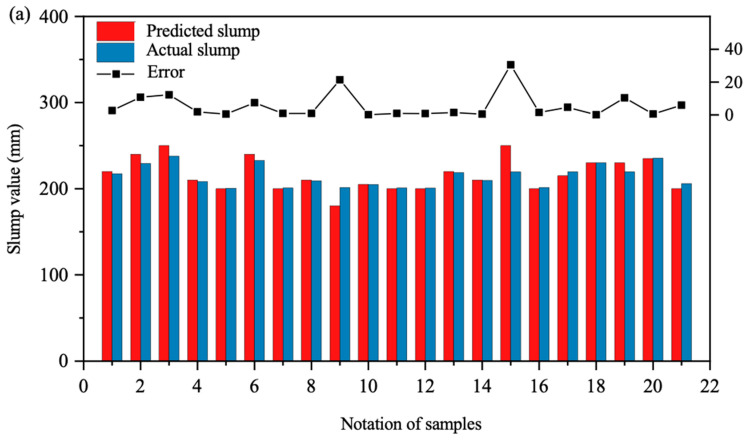

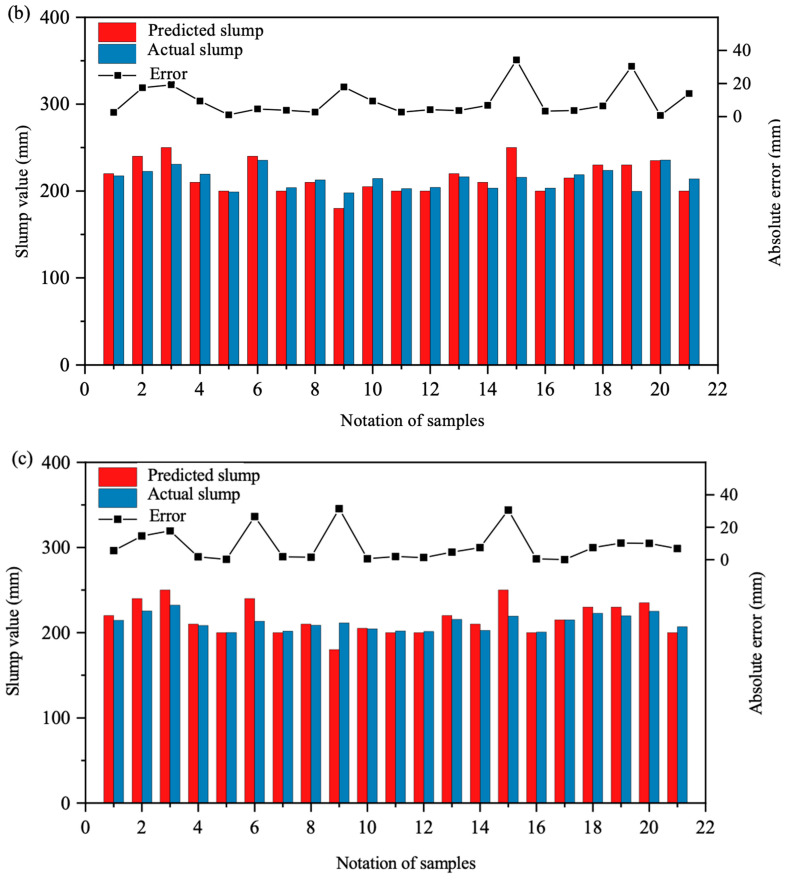
Error analyses between the predicted value and actual value of the slump of machine learning model: (**a**) BPNN model prediction error; (**b**) SVM model prediction error; (**c**) RF model prediction error.

Figure 10 presents an error analysis of the model predictions. Subfigure (a) details the error between predicted and actual slump values for BPNN on the validation set, (b) for SVM, and (c) for RF. The histograms represent the distribution of predicted and actual slump values within the validation set, while the line graphs depict the absolute errors between model outputs and actual values. From this analysis, it is apparent that subgraph (c) exhibits narrower histogram intervals and a lower error curve, indicating that RF has the smallest absolute errors between the predicted and actual values in the validation set. In contrast, subgraph (a) shows slightly wider histogram intervals and a moderately higher error curve than subgraph (c), while subgraph (b) displays the widest histogram intervals and the highest error curve, indicating the largest absolute errors for SVM. Thus, RF demonstrates the highest predictive accuracy in the validation set, followed by BPNN, with SVM exhibiting the largest deviations.

**Figure 11:**
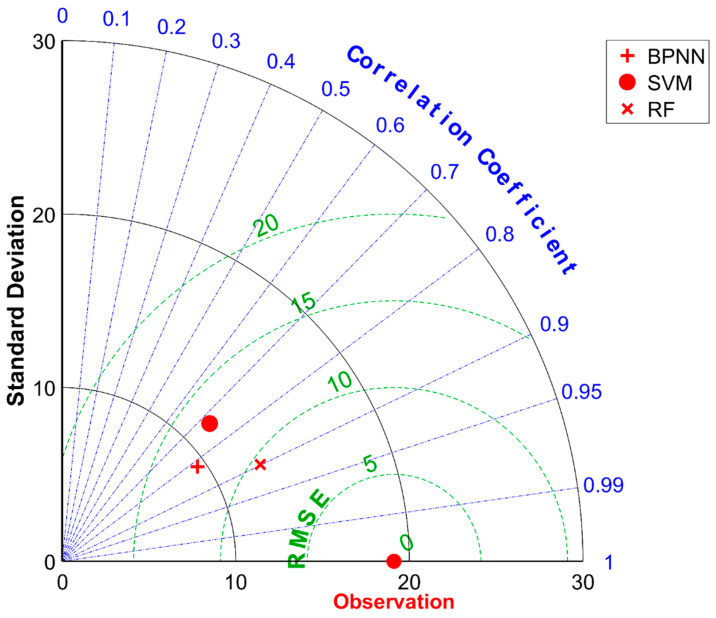
Taylor diagram comparison of the machine learning model validation set’s prediction performance.

Figure 11 presents the Taylor diagram for evaluating the accuracy of three predictive models on the validation dataset. In this diagram, the scatter points represent machine learning models, the concentric arcs centered at the origin depict the standard deviation (SD), the radial lines signify the correlation coefficient (R), and the arcs centered around the reference point (observed data) indicate the Root Mean Square Error (RMSE). The ideal scenario, where the observed point is located at R = 1 and RMSE = 1, serves as a benchmark. Notably, the standard deviation SD = 19.1143 corresponds to the variability in the slump samples of the validation set.

By analyzing the positional relationships between the scatter points and the radial lines, it becomes evident that the SVM (Support Vector Machine) model for slump prediction in the validation set exhibits a correlation coefficient R of less than 0.8. This indicates a moderate, rather than strong, correlation between the predicted and actual values, thus failing to meet the modeling requirements. Further, by comparing the angles formed by the radial lines (emanating from the origin towards each scatter point) with the vertical axis, we can deduce the performance ranking as RRF (Random Forest Regression) > RBPNN (Radial Basis Function Neural Network) > RSVM (SVM’s correlation coefficient).

Examining the straight-line distances from each scatter point to the observed point reveals that BPNN (Back Propagation Neural Network) achieves the closest proximity, followed by SVM, with RF being the furthest. This translates into a performance order in terms of RMSE as RMSE_RF < RMSE_BPNN < RMSE_SVM, indicating that RF provides the most accurate predictions in terms of minimizing the Root Mean Square Error. Consequently, Random Forest Regression (RRF) is selected as the predictive model for concrete slump in this study, aimed at facilitating multi-objective optimization of mix proportions. The trained model is henceforth referred to as MODEL. This choice is based on its superior performance in minimizing prediction errors and maximizing correlation with observed data, thereby enhancing the reliability and applicability of the model in practical concrete production scenarios.

## 4. Conclusions

In the end, this study primarily delves into the fundamental principles of the artificial intelligence algorithms employed and outlines the process of determining the predictive models for concrete compressive strength and slump. The process commences with the meticulous screening, analysis, and preprocessing of the collected data. Subsequently, Particle Swarm Optimization (PSO) is utilized for hyperparameter tuning, individually optimizing the parameters of the Back Propagation Neural Network (BPNN), Support Vector Machine (SVM), and Random Forest (RF) models before subjecting them to training. Finally, a Taylor diagram is employed to compare the predictive accuracy of the trained BPNN, SVM, and RF models, thereby identifying the model with the optimal performance.

The outcomes of the hyperparameter optimization reveal that significant variations exist in the optimal hyperparameters for the same machine learning model when applied to predicting different output variables. This underscores the necessity of modeling distinct properties of concrete performance separately, emphasizing the specificity required in predicting diverse concrete attributes. While all three models demonstrate high predictive accuracy within the validation set, they exhibit discernible differences in terms of Root Mean Square Error (RMSE) and correlation coefficient (R). The comparative analysis concludes that the optimized BPNN model outperforms in compressive strength prediction, while the optimized RF model excels in slump prediction. Consequently, the BPNN model, taking concrete mix proportions as input and compressive strength as output, is denoted as NET. Similarly, the RF model, with concrete mix proportions as input and slump as output, is referred to as MODEL. This distinction underscores the tailored nature of each model in addressing specific performance metrics of concrete.

## Figures and Tables

**Table 1:** Statistics of the total training sample set.

Variables	Minimum	Maximum	Average	Median	S.D.
C_C_ (kg)	120	366	188.74	155.50	67.25
C_FLA_ (kg)	0	260	108.11	100	54.05
C_MP_ (kg)	0	193	77.58	81	36.52
C_FA_ (kg)	600	920	763.65	775.50	60.16
C_CA_ (kg)	747	1130	1005.54	1040	92.26
C_W_ (kg)	130	237	178.82	175	20.98
C_SP_ (kg)	3	19.50	7.19	6.25	2.67
UCS (MPa)	17.19	74.90	39.76	35.45	12.96
slump (mm)	155	250	209.88	200	17.38

Note: S.D. stands for standard deviation.

**Table 2:** Training parameters of BPNN.

Training Function	TRAINLM
Transfer function of hidden layer	logsig
Transfer function of output layer	purelin
Learning rate	0.0001
Desired minimum error	10^−5^
Maximum number of training epochs	2000

**Table 3:** Value range of the hyperparameters.

Machine Learning Models	Hyperparameters	Optimization Results
BPNN	Number of hidden layers	[1, 4]
	Number of neurons in hidden layer	[1, 30]
SVM	c	[0.1, 10,000]
	Gamma	[0.0001, 0.001]
RF	Number of trees in the forest	[1, 50]
	Minimum samples required to split a leaf node	[1, 5]

**Table 4:** The best RMSE of the validation set.

Models	BPNN	SVM	RF
RMSE (MPa)	6.2965	6.7231	6.8972

**Table 5:** Results of hyperparameter optimization of the strength of the prediction model.

Machine Learning Models	Hyperparameters	Optimization Results
BPNN	Number of hidden layers	1
	Number of neurons in hidden layer	17
SVM	c	1904.148
	Gamma	0.01
RF	Number of trees in the forest	24
	Minimum samples required to split a leaf node	4

**Table 6:** Predict performance evaluation of the ML model.

Models		Evaluation Metrics		
		R	RMSE (MPa)	MAE (MPa)
BPNN	Training set	0.9531	4.2568	2.6627
	Validation set	0.9528	4.2183	3.4193
SVM	Training set	0.8980	5.6187	2.9439
	Validation set	0.8871	7.2108	5.7156
RF	Training set	0.7401	8.9797	6.5097
	Validation set	0.8546	7.3925	6.3588

**Table 7:** The best RMSE of the validation set.

Model	BPNN	SVM	RF
RMSE (mm)	11.5789	13.0015	8.3192

**Table 8:** Results of hyperparameter optimization of the slump prediction model.

Machine Learning Models	Hyperparameters	Optimization Results
BPNN	Number of hidden layers	2
	Number of neurons in hidden layer	[10, 14]
SVM	c	30.01
	Gamma	0.01
RF	Number of trees in the forest	36
	Minimum samples required to split a leaf node	4

**Table 9:** Model validation set performance evaluation.

Models		Evaluation Metrics		
		R	RMSE (mm)	MAE (mm)
BPNN	Training set	0.8317	9.8379	5.8692
	Validation set	0.8194	13.0695	8.6892
SVM	Training set	0.7312	11.8763	7.1734
	Validation set	0.7301	9.4906	9.4150
RF	Training set	0.9540	6.6064	4.1958
	Validation set	0.8986	9.4906	5.5034

## Data Availability

Data will be available on request.
